# Safety of preoperative branch embolization in patients undergoing evar

**DOI:** 10.1590/1677-5449.202201372

**Published:** 2024-02-23

**Authors:** Luis Ángel Suárez González, Iñigo Lozano Martínez-Luengas, Pablo del Canto Peruyera, Manuel Javier Vallina-Victorero Vazquez

**Affiliations:** 1 Hospital Universitario de Cabueñes, Gijón, España.

**Keywords:** EVAR, inferior mesenteric artery, lumbar arteries, embolization, EVAR, artéria mesentérica inferior, artérias lombares, embolização

## Abstract

The purpose of this systematic review is to evaluate the safety of pre-endovascular abdominal aortic aneurysm repair (EVAR) embolization of aortic side branches - the inferior mesenteric artery and lumbar arteries. Preferred Reporting Items for Systematic Reviews and Meta-Analysis guidelines were followed. A search of MEDLINE and DIMENSION databases identified 9 studies published from 2011 to 2021 that satisfied the inclusion and exclusion criteria. These studies were analyzed to detect the incidence of embolization-related complications. A total of 482 patients underwent preoperative aortic side branch embolization, 30 (6.2%) of whom suffered some kind of minor complication. The only major complication observed was ischemic colitis in 4 (0.82%) patients, two (0.41%) of whom died after bowel resection surgery. Regarding these findings, aortic side branch embolization seems to be a safe procedure, with very low percentages of both minor and major complications.

## Resumo

O objetivo desta revisão sistemática foi avaliar a segurança da embolização de artéria mesentérica inferior (AMI) e artérias lombares (ALs) pré-correção endovascular de aneurisma da aorta abdominal. Foram realizadas pesquisas nas bases de dados MEDLINE e Dimensions. Foram encontrados 9 estudos publicados de 2011 a 2021 que atendiam aos critérios de inclusão e exclusão. Os estudos foram analisados ​​para definir a incidência de complicações relacionadas à embolização. No total, 482 pacientes foram submetidos a embolização de AMI e/ou ALs, dos quais 30 (6,2%) sofreram algum tipo de complicação menor. A única complicação importante observada foi colite isquêmica em 4 (0,82%) pacientes. Dois (0,41%) desses pacientes morreram após cirurgia de ressecção intestinal. Em relação a esses achados, a embolização de AMI e ALs parece ser um procedimento seguro, com um percentual muito baixo de complicações menores e importantes.

## INTRODUCTION

Since first performed by Parodi in 1990,^1^ endovascular aortic repair (EVAR) has become the predominant technique for treating aortic aneurysms because it is associated with lower morbidity and mortality compared with open surgical repair (OSR).^2^

However, EVAR also has some weak points compared with OSR, mainly the need for a greater number of reinterventions, which can be linked with sac diameter enlargement and aneurysm-related complications.^3,4^ These reinterventions are primary linked to endoleaks, and the majority are caused by type 2 endoleaks (T2E). T2E can account for 50% of all endoleaks,^5^ and recent studies point to incidence percentages as high as 17% of all EVAR.^6^

T2E are defined as backflow into the aneurysm sac from aortic side branches (ASB), mainly the inferior mesenteric artery (IMA) or the lumbar arteries (LA). The relationships between these vessels and the origin of T2E have been analyzed and the preoperative permeability^7^ and diameter of the IMA and the number of patent LA^8^ have been identified as risk factors for T2E. When T2E occur, they can be very difficult to manage, increasing the risk of reinterventions and EVAR-related complications during follow-up.

Because of this, preemptive ASB embolization has been proposed to reduce the incidence of T2E. Great technical success has been reported with these procedures. However, serious complications, such as ischemic colitis (IC)^9,10^ or spinal cord ischemia^11,12^ can occur when these vessels are embolized. The aim of this systematic review is to analyze the incidence of complications related to preemptive ASB embolization.

## MATERIAL AND METHODS

### Literature search

We systematically reviewed original articles regarding preoperative aortic side branch embolization in patients undergoing EVAR, using the Preferred Reporting Items for Systematic Reviews and Meta-Analysis (PRISMA) methodology (http://www.prisma-statement.org).

A systematic literature search was performed on the MEDLINE and DIMENSIONS databases. Keywords used for searching were (“inferior mesenteric artery” OR “lumbar artery” OR “lumbar arteries” OR “aortic side branches”) AND (“embolization” or “occlusion”) AND (“aneurysm” or “EVAR”). Scientific papers published from 2011 to 2021 were included.

### Study selection

Inclusion criteria were studies analyzing the role of preoperative ASB embolization to decrease the risk of T2E in patients who underwent EVAR. Studies not including information about clinical complications after the procedure were excluded.

Title and abstract screenings and full text analysis were performed by two reviewers. When there were discrepancies between the reviewers’ decisions, disagreements were resolved by consensus.

### Study endpoints

The primary endpoint of this study was to asset the security of preventive embolization of ASB in patients undergoing EVAR.

Secondary endpoints include description of technical aspects of embolization: timing of embolization, embolization access site, and embolization material.

## RESULTS

Nine studies from an initial number of 1512 titles identified met the criteria for inclusion in this systematic review. Two of these studies were retrospective case series and the remaining seven studies were retrospective cohort studies.

The search strategy and exclusions were in accordance with the PRISMA framework.^13^

A total of 482 patients underwent IMA or LA embolization. IMA was the target vessel in five studies, while both IMA and LA were the targets in the other five studies.

### Embolization technique

The preferred access site for embolization was the common femoral artery. Alternatively, brachial access was used, especially if the IMA had acute angulation and presented anatomic catheterization difficulties.

Pre-EVAR embolization was the chosen timing for the procedure in five studies. Another three studies preferred to perform embolization at the same time as EVAR. The remaining study mixed pre-EVAR and during EVAR embolization, left to operator choice. When pre-EVAR embolization was chosen, the procedures were performed under local anesthesia.

When the IMA was the target vessel, the studies consistently emphasize that occlusion should be performed prior to the origin of the left colic artery as a measure to decrease the risk of mesenteric ischemic complications. In LA embolization, occlusion at the ostium is recommended to avoid spinal ischemia.

Embolization was performed using coils in five studies and using vascular plugs in two studies. The remaining three studies used both vascular plugs and coils to achieve successful ASB embolization. One of the studies specifically mentions that chemoembolization agents and embolization particles should be avoided because their use could be associated with distal migration and consequent ischemic complications. The Amplatzer vascular Plug 4 is a low profile plug compatible with 0.038” guide wire compatible diagnostic catheters that allows distal vasculature to be reached through tortuous anatomy^14^ and was the plug chosen in four studies. The remaining study using vascular plugs does not mention which specific type of plug was used.

Embolization techniques are summarized in Table 1

**Table 1:** Embolization technique details.

Author (year)	Access site	Embolization method	Timing of embolization
Burbelko et al.^15^	Transfemoral	Amplatzer Vascular plug	Pre-EVAR (1-4 weeks)
Aoki et al.^16^	Transfemoral	Coils	During EVAR
Alerci et al.^17^	Transfemoral	Coils	Pre-EVAR (15 days)
Petit et al.^18^	Transfemoral	Coils, Amplatzer Vascular plug	During EVAR
Branzan et al.^19^	Transfemoral	Vascular plug, coils	Pre-EVAR
			During EVAR
	Transbrachial (if acute downward angulation of the IMA)		
Müller-Wille et al.^20^	Transfemoral	Amplatzer Vascular plug	Pre-EVAR
	Transbrachial		
Ward et al.^21^	Transfemoral	Coils	Pre-EVAR (4-8 weeks)
Vaillant et al.^22^	Transfemoral	Coils (IMA <5 mm)	Pre- EVAR (24-72h)
		Amplatzer Vascular plug (IMA <5mm)	
Manunga et al.^23^	Transfemoral	Coils	During EVAR

### Safety

Five of these studies did not report any complications related with ASB embolization or the procedure. Minor complications were reported in four studies, with 30 patients (6.2%) affected. These minor complications included 4 (0.8%) patients with local hematoma at the puncture site, 10 patients (2.1%) who suffered from abdominal pain due to IMA embolization, and 16 (3.3%) who had moderate back pain related to muscle ischemia associated with LA embolization. Patients with back pain were treated using nonsteroidal anti-inflammatory analgesics and none of them developed any neurological deficits. Regarding the patients with abdominal pain, all of them had sigmoidoscopy and all were negative for ischemic changes. They were treated with intravenous hydration with relief of symptoms.

Major complications were reported in 4 patients (0.8%), all of them suffering from IC. Three (75%) of these four patients underwent bowel resection. Despite surgery, two (50%) of IC patients died in the postintervention period, resulting in a total mortality rate of 0.41% in patients undergoing ASB embolization.

Summarized results of the studies included in this review are shown in Table 2.

**Table 2:** Results and data from the studies included in the systematic review.

Author	Level of evidence	Patients (n)	Type of study	Target vessels	Minor complications	Major complications
Burbelko et al.^15^	2b	40	Retrospective cohort	IMA, LA	0	0
Aoki et al.^16^ (2017)	2b	24	Retrospective cohort	IMA, LA	0	0
Alerci et al.^17^	2b	39	Retrospective cohort	IMA, LA	0	0
Petit et al.^18^	2b	52	Retrospective cohort	IMA	0	2 IC
Branzan et al.^19^	4	139	Retrospective case series	IMA, LA	16 moderate back pain	1 IC (1 death)
Müller-Wille et al.^20^	4	31	Retrospective case series	IMA	2 hematoma	0
Ward et al.^21^	2b	108	Retrospective cohort	IMA	10 abdominal pain	1 IC (1 death)
Vaillant et al.^22^	2b	37	Retrospective cohort	IMA	2 hematoma	0
Manunga et al.^23^	4	12	Retrospective cohort	IMA	0	0

## DISCUSSION

Type 2 endoleaks remain the major problem after EVAR procedures. Their relationship with ASB has been well described in the literature.^24-26^ Preoperative patency of the IMA (and its diameter) and the number of preoperative patent LA^27,28^ have been identified as risk factors for T2E development in patients undergoing EVAR. Current guidelines recommend treating T2E when they appear after EVAR and sac enlargement of 5-10 mm is detected during follow-up.^29,30^

Once established, T2E constitute a challenging problem to deal with and treatment after development does not seem to have high efficacy. Ultee et al.^31^ found that sac growth was common after treatment of T2E and rupture rates (<2%) were not significantly different from those reported in post-EVAR studies in general. These data are supported by a recent study by the ODISSEUS group.^6^ This study found no difference in overall survival between patients who underwent a secondary intervention to treat T2E and those who were treated conservatively, and sac growth was observed in 93.1% after secondary intervention.

Because of these discouraging results for T2E treatment post-development, preoperative ASB embolization has been proposed to decrease the risk of T2E, using both coils and vascular plugs, with great technical success. Various studies suggest the efficacy of these procedures for reducing development of T2E, number of reinterventions, and sac growth after EVAR.^32^ An ongoing multicenter randomized trial will try to clarify the efficacy of preoperative IMA embolization.^33^

Despite the findings of these studies, preemptive ASB embolization has still not been standardized. This could be because of certain disadvantages associated with the procedure: increased use of fluoroscopy and increased intervention times, increased radiation dosage and contrast usage, and higher operating costs. However, the main concern relating to standardizing these interventions prior to an EVAR could be the complications related to the embolization procedure.

In this systematic review, we found a very low number of complications (n= 34, 6%) related to ASB embolization. Most of the complications (n=30, 5%) could be considered minor and were solved in a few days with conservative and local medical treatment (local hematoma, diffuse pain) with no other interventions needed.

Regarding major complications, ischemic colitis was the only major complication reported, seen in a very low percentage of patients (0.7%) and with an even lower incidence of mortality (only 2 deaths, equivalent to 0.35%). Remarkably, no spinal cord ischemia was described. This might be surprising because this kind of neurological complications had been previously described when embolization procedures are performed to treat T2E emerging after EVAR.^11,34^

Ischemic colitis has been one of the biggest causes of morbidity and mortality associated with aortic surgery since its beginnings. This applies to both OSR and EVAR.

When we analyze OSR complications, IC has documented incidence rates that vary from 5-17.5% of patients undergoing abdominal aortic aneurysm surgery.^35,36^

Patients undergoing EVAR are not exempt from IC. The incidence of IC in these procedures is above 2%, which is lower than the rates observed after OSR. Despite this lower rate of IC, statistical significance has not been found regarding IC after OSR or EVAR.^37^

Almost half of the patients that suffer from postoperative IC require bowel resection surgery. However, despite these interventions, patients’ postoperative course once IC is established is quite unlucky and mortality exceeds 50%.

If we analyze the need for resection surgery and mortality after IC in patients with preoperative ASB embolization, we find similar rates to those seen in patients suffering from IC after OSR and EVAR. However, the incidence of IC after embolization procedures is clearly lower than after treatment of AAA, whether by OSR or by EVAR.

The embolization materials preferred in the studies analyzed were coils and plugs, specifically the Amplatzer Vascular Plug 4. It might be surprising to find that none of these authors used chemoembolization agents or embolization particles, especially considering the increasing popularity of these agents and their use in embolization procedures. However, a review of the literature shows that a great number of documented ischemic complications after treatment of an already established T2E are related with the use of these materials as embolic agents.^9-12,38^ Moreover one of the most recent studies, published by Branzan et al.,^19^ states that these agents were strictly prohibited because they could cause non-target ischemic damage. Therefore, the very low rate of ischemic complications found in this systematic review could be related to the use of appropriate embolization materials and avoiding use of agents that could increase the risk of ischemia due to migration.

Based on the literature, coils and vascular plugs should be used as embolization materials to decrease the risk of these ischemic complications, avoiding chemoembolization agents or embolization particles, because the latter could impose a greater risk of non-target ischemic events. When the IMA is the target vessel, embolization materials should be placed before the origin of the left colic artery. If the treated vessels are LA, recommendations suggest performing embolization at the ostium to avoid risk of spinal cord ischemia.

## CONCLUSIONS

Preventive ASB embolization is a technique with a very low percentage of complications. When complications are seen, most of them could be described as minor, with a very low rate of procedure-related ischemic events, even lower than those reported after standard EVAR. When IC occurs, rates of need for bowel resection and mortality are similar to those seen in patients with IC after OSR or EVAR.

However, it is necessary to report on larger series and conduct RCTs to confirm the safety profile of preemptive aortic branch embolization, since many of the studies analyzed in this review have low evidence levels. Larger sample sizes would not only enable us to perform these procedures with greater security and higher evidence levels, but would also yield more techniques for performing them more effectively.
